# “Negative Vaccination” by Specific CD4^+^ T Cell Tolerisation Enhances Virus-Specific Protective Antibody Responses

**DOI:** 10.1371/journal.pone.0001162

**Published:** 2007-11-14

**Authors:** Karl S. Lang, Ahmed N. Hegazy, Philipp A. Lang, Bruno Eschli, Max Löhning, Hans Hengartner, Rolf M. Zinkernagel, Mike Recher

**Affiliations:** Institute of Experimental Immunology, Department of Pathology, University Hospital Zurich, Zurich, Switzerland; Federal University of Sao Paulo, Brazil

## Abstract

**Background:**

Cooperation of CD4^+^ T helper cells with specific B cells is crucial for protective vaccination against pathogens by inducing long-lived neutralizing antibody responses. During infection with persistence-prone viruses, prolonged virus replication correlates with low neutralizing antibody responses. We recently described that a viral mutant of lymphocytic choriomeningitis virus (LCMV), which lacks a T helper epitope, counterintuitively induced an enhanced protective antibody response. Likewise, partial depletion of the CD4^+^ T cell compartment by using anti-CD4 antibodies enhanced protective antibodies.

**Principal Findings:**

Here we have developed a protocol to selectively reduce the CD4^+^ T cell response against viral CD4^+^ T cell epitopes. We demonstrate that *in vivo* treatment with LCMV-derived MHC-II peptides induced non-responsiveness of specific CD4^+^ T cells without affecting CD4^+^ T cell reactivity towards other antigens. This was associated with accelerated virus-specific neutralizing IgG-antibody responses. In contrast to a complete absence of CD4^+^ T cell help, tolerisation did not impair CD8^+^ T cell responses.

**Conclusions:**

This result reveals a novel “negative vaccination” strategy where specific CD4^+^ T cell unresponsiveness may be used to enhance the delayed protective antibody responses in chronic virus infections.

## Introduction

Induction of a long-lived protective neutralizing IgG response is a hallmark of virtually all successful vaccinations [1]. However, vaccination strategies against many important human pathogens have failed so far. These include vaccination against HIV [2], HCV [3], malaria [4] and tuberculosis [5], all representing chronic persisting infections. Vaccination failure correlates with much delayed and often poor pathogen-specific protective antibody responses [6], [7] on one side and often with great variability of the protective antigen on the other side. The delayed neutralizing antibody response against the noncytopathic lymphocytic choriomeningitis virus (LCMV) in mice correlates with low precursor frequencies of B cells specific for the neutralizing antigenic site [8], with mutational variability of the relevant glycoprotein determinant [9] and with CD8^+^ T cell-mediated immunopathology [10]. In addition, LCMV and several persisting human pathogens like HCV [11] and HIV [12] induce a T helper cell-dependent, mostly polyclonal B cell activation [13] whereas protective antibodies specific for the virus surface glycoprotein remain undetectably low for more than 50–100 days. Counter-intuitively, experimental partial–but not complete-reduction of T helper cell responses reduced polyclonal B cell activation and enhanced virus-specific neutralizing antibody responses [14]. Consistently, transfer of CD27-competent T helper cells into CD27-deficient mice reduced the improved virus-neutralizing antibody titers observed after LCMV infection of these mice [15]. Both experiments suggested that too much T help somehow impairs virus-neutralizing antibody responses. Here we show that “negative vaccination” by specific tolerisation with LCMV MHC-class II-restricted CD4^+^ T cell epitopes inhibited virus-specific helper CD4^+^ T cell responses but enhanced protective antibody responses in terms of earlier onset and higher titers without impairing protective CD8^+^ T cell responses or third-party specific immune responses.

## Results

### Peptide tolerisation leads to functional impairment of virus-specific CD4^+^ T cells

**Figure 1:**
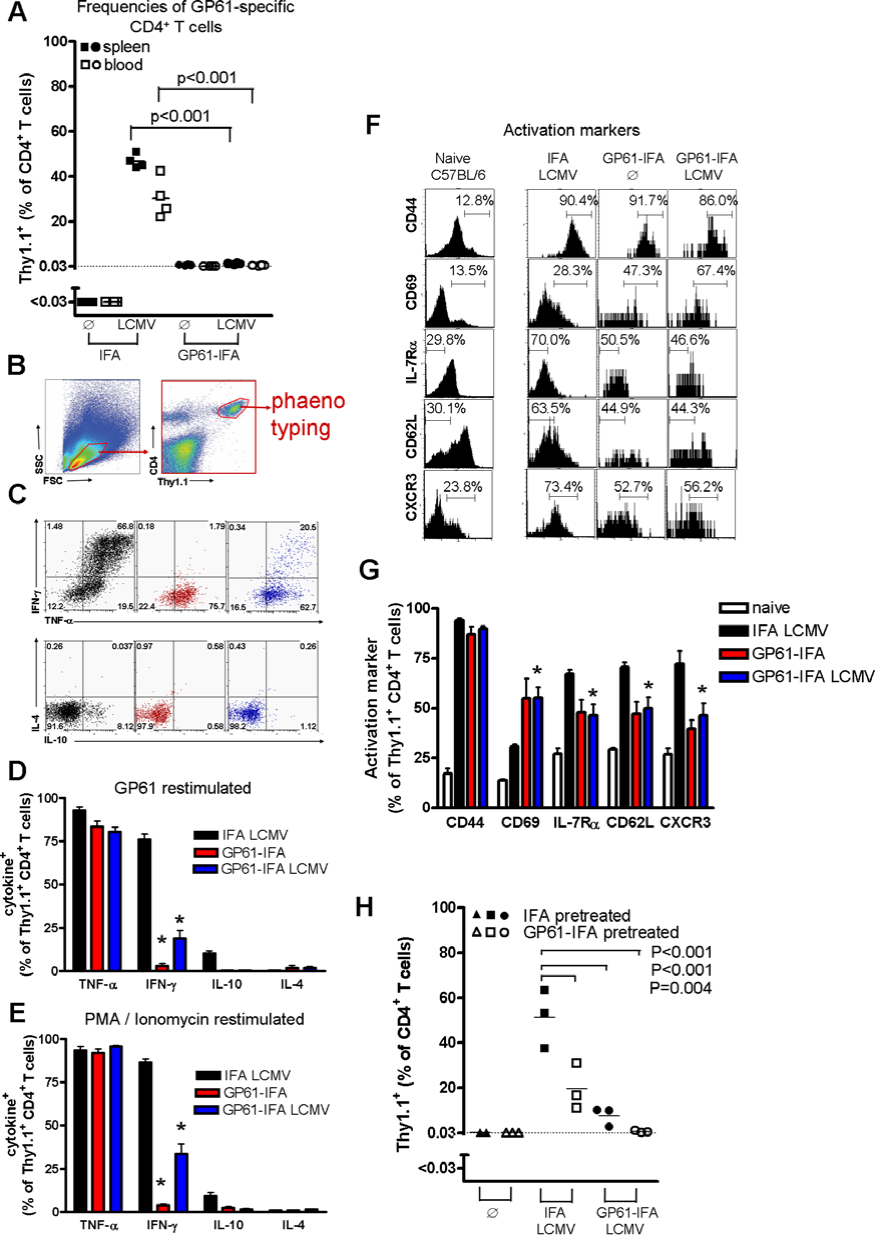
Peptide tolerisation leads to functional impairment of virus-specific CD4^+^ T cells. A–G: 5×10^4^ splenocytes from mice transgenic for a T cell receptor recognizing the LCMV helper epitope GP61 (LCMV-glycoprotein_61-80_/I-A^b^-specific TCR, SMARTA mice) and expressing the T cell marker Thy1.1 were adoptively transferred into C57BL/6 mice on day -10. One group of mice was treated with 100 µg GP61 dissolved in IFA (squares), while control mice were treated with IFA alone (circles) at days -9, -6, -3. At day 0 mice were infected with 200pfu LCMV-WE or left untreated. Seven days after infection mice were analyzed for CD4^+^ T cell function. (A) Frequencies of GP61-specific CD4^+^ T cells (Thy1.1^+^ T cells) were analysed in spleen and blood. (B) For further phenotyping of thy1.1^+^ CD4^+^ cells, cells were gated as shown in the gating tree. (C) Cells were re-stimulated with GP61 *in vitro* and after six hours Thy1.1^+^ T cells were analysed for intracellular expression of IL-4, IL-10, IFN-γ and TNF-α by FACS analysis (one of four representative dot blots is shown. (D) Bar charts show data analyzed in C (n = 4,*p<0.001). (E) Cells were re-stimulated with PMA/Ionomycin in vitro and after six hours Thy1.1^+^ T cells were analyzed for intracellular expression of IL-4, IL-10, IFN-γ and TNF-α by FACS analysis (n = 4, *p<0.001). (F) GP61-specific Thy1.1^+^ CD4^+^ T cells and CD4^+^ T cells from untreated C57BL/6 mice were analysed for the activation markers CD69, IL-7Rα, CD62L, CD44 and CXCR3 (marker is set on the activated phenotype). (G) Statistically analysis from the data derived in F (n = 4, *p<0.05). H: 5×10^6^ splenocytes from mice transgenic for a T cell receptor recognizing the LCMV helper epitope GP61 (LCMV-glycoprotein_61-80_/I-A^b^-specific TCR, SMARTA mice) and expressing the T cell marker Thy1.1 were transferred into a total of six C57BL/6 mice on day -11. Three of those mice were treated with 100 µg GP61 dissolved in IFA (GP61-IFA-pretreated), while the other three mice were treated with IFA alone (IFA-pretreated) at days -10, -7, -4. In addition six C57BL/6 mice were treated with 100 µg GP61 dissolved in IFA (GP61-IFA-treated) and six control mice were treated with IFA alone (IFA-treated) at days -10, -7, -4. At day -1 splenocytes from each of the three GP61-IFA-pretreated mice were transferred into one GP61-IFA-treated, one IFA-treated and one untreated C57BL/6 mouse (one untreated C57BL/6 mouse died during transfer). In parallel splenocytes from each IFA-pretreated mouse was transferred into one GP61-IFA-treated, one IFA-treated and one untreated C57BL/6 mouse (transfer scheme Figure S2). Number of transferred splenocytes was adapted to the frequencies so that all mice received the same numbers of Thy1.1^+^ CD4^+^ T cells at day -1 (data not shown). On day 0 mice were infected with 200pfu LCMV-WE. Six days after infection mice were analyzed for Thy1.1^+^ CD4^+^ T cells by FACS analysis.

Exposure to and persistence of sufficient antigen can lead to initial over-activation and subsequent exhaustion of CD8^+^ T cells [16]. This phenomenon is commonly observed during persistent viral infection [16], and can be mimicked by administration of synthetic peptide-antigen by continuous treatment or slow release in incomplete Freund̀s adjuvant (IFA) [17]. Naïve or memory CD8^+^ T cells undergo rapid proliferation after contact with antigen in IFA, followed by apoptosis and deletion [17]. Similarly, administration of CD4^+^ T helper cell peptide in IFA intraperitoneally can inhibit onset of CD4 T cell dependent autoimmune disease [18]. LCMV-specific peptide tolerisation of CD4^+^ T cell helper function was analyzed by adoptively transferring 5×10^4^ indicator splenocytes from a mouse transgenic for a T cell receptor recognizing the LCMV helper epitope GP61 (LCMV-glycoprotein_61-80_/I-A^b^-specific TCR, SMARTA mice) into naïve C57BL/6 mice. Transferred T cells expressed the T cell marker Thy1.1 and thus could be tracked by FACS analysis in the Thy1.2-expressing C57BL/6 mouse recipients. We transferred as few as 5×10^4^ splenocytes which kept the precursor frequency in a physiological range and cells were still detectable by FACS. Recipient mice were treated with 100 µg GP61 in IFA on days -9, -6, -3 before infection with LCMV. A control group of recipient mice was treated with IFA alone. IFA treatment without peptide did not expand transfused Thy1.1^+^ T cells, demonstrating that IFA alone did not activate CD4^+^ T cells through bystander activation (Figure 1A). T helper cells from peptide non-treated mice displayed a strong expansion upon LCMV infection (>1000 fold, Figure 1A), whereas T helper cells from peptide treated mice were few and showed no expansion (<2 fold, Figure 1A), implying functional non-responsiveness of those T cells. We compared the phenotype of the few remaining GP61-IFA treated T cells with the peptide non-treated vastly expanded T cells by gating on GP61 specific Thy1.1^+^ T cells (Figure 1B). Production of the Th1 cytokine IFN-γ was strongly reduced in GP61-IFA treated Thy1.1^+^ T cells compared to IFA treated T cells, whereas TNF-α production was within normal range (Figure 1B–E). Peptide-treatment did not shift the effector phenotype of T helper cells towards Th2, as secretion of IL-4 and IL-10 was low (Figure 1B–E). Thus, peptide-induced T helper cell non-responsiveness affected antigen-specific T helper cells and remained robust even after LCMV infection. Functional non-responsiveness of T cells, despite physical presence of very few T cells as measured here, has been described as T cell “anergy” , associated with a disturbed intracellular signaling of diacylglycerol [19]. Such anergy can be experimentally bypassed *in vitro* by stimulation of T cells with PMA and ionomycin, which directly activate proteinkinase C and increase intracellular calcium flux. After stimulation with PMA plus ionomycin, Thy1.1^+^ T cells from control mice displayed strong production of IFN-γ, however tolerised T helper cells virtually did not respond to this direct activation stimulus (Figure 1E). Thus, treatment with GP61-IFA induced a non-responsiveness that was rather complete and not mediated by “classical” anergy.

To further characterise specific T helper cell non-responsiveness, several activation markers were screened which are regulated upon TCR triggering in a chronological program. Very early after activation, CD4^+^ T cells are known to up-regulate CD44, which remains up-regulated. A transient up-regulation of CD69 is followed by down-regulation of IL-7R and CD62L. Depending on their differentiation some of these T cells up-regulate CXCR3 and/or down-regulate CCR7 later on [20]–[22]. Those few GP61-specific CD4^+^ T cells detected in the spleen after GP61-IFA treatment showed similar expression of CD44 compared to peptide non-treated T helper cells, indicating that they were stimulated by antigen (Figure 1F&G). They displayed a significantly higher expression of the activation marker CD69 after LCMV infection compared to control IFA-only treated T helper cells while down-regulation of IL-7Rα and CD62L was significantly less pronounced in GP61-IFA treated T helper cells (Figure 1E&F). CXCR3 was expressed at significantly lower levels on GP61-IFA treated T cells (Figure 1E&F), while CCR7 was not expressed differently on GP61-tolerized T cells (data not shown). Therefore, we concluded that the very few remaining GP61-IFA treated CD4^+^ T cells were sufficiently activated to up-regulate the effector marker CD44, but stayed in an early activation status without differentiation into full-blown effector T helper cells, which probably was the reason for the observed absence of proliferation.

PD-1 has been demonstrated to be a marker expressed by exhausted CD8^+^ T cells in LCMV as well as in HIV infection [23]. Additionally, prolonged antigen contact may induce a regulatory phenotype in CD4^+^ T cells. However, we did not find any difference between IFA-only and GP61-IFA treated CD4^+^ T cells with respect to PD-1 expression or expression of classical regulatory T cell phenotype markers (Figure S1).

Next, the maintenance of non-responsiveness following T helper cell peptide treatment was analyzed. Therefore 5×10^6^ indicator splenocytes were transferred from SMARTA×Thy1.1^+^ mice into C57BL/6 recipients. Mice were treated with GP61-IFA, and control mice were treated with IFA alone. Nine days later, the spleen cells were transferred into new IFA or GP61-IFA treated mice and expansion was analyzed after infection with LCMV WE (scheme of transfer: Figure S2). We found that the presence of GP61-IFA in the secondary recipient mouse completely inhibited expansion of Thy1.1^+^ cells (Figure 1H). Peptide-tolerised T helper cells proliferated to a much reduced extent in peptide-free IFA-only treated second recipients after virus infection (Figure 1H), indicating a partially reversible intrinsic non-responsiveness of the transferred GP61-specific T cells. In conclusion, specific peptide treatment drastically reduced specific CD4^+^ T helper cell proliferation. The few remaining cells were seemingly “stuck” in an early (probably transient) activation state and/or were preferentially undergoing cell death during the subsequent activation steps.

### Peptide-induced helper T cell non-responsiveness improves LCMV-specific neutralizing antibody responses

**Figure 2:**
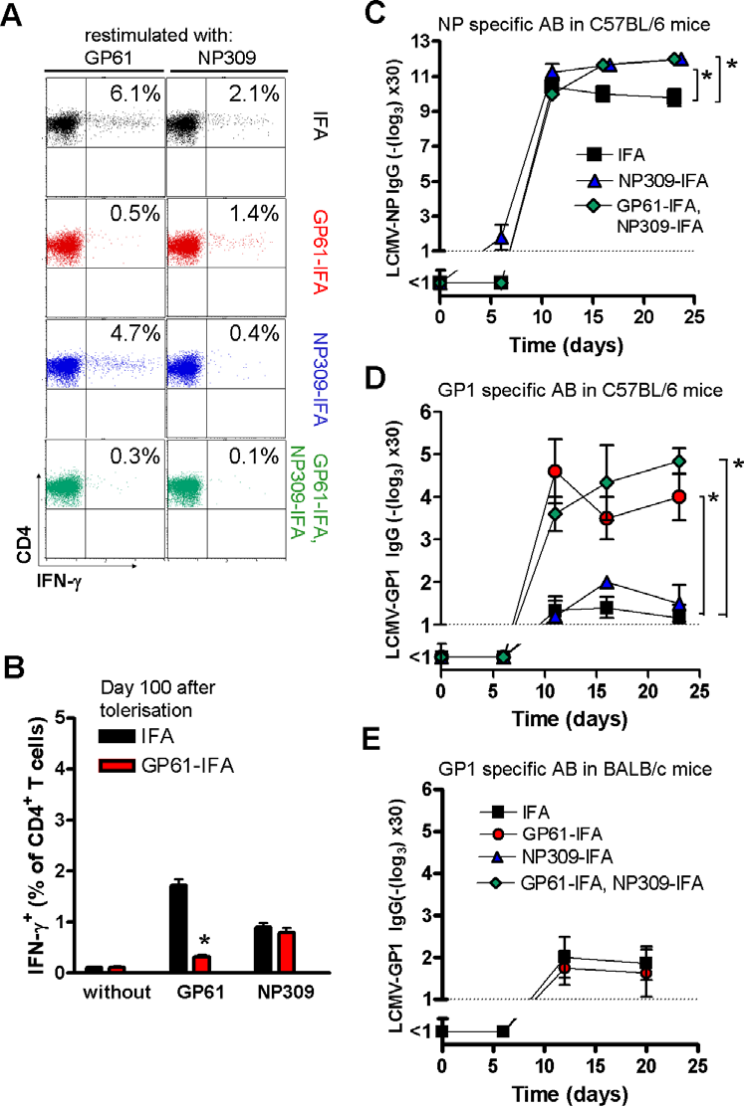
Tolerisation of CD4^+^ T cells improves LCMV-specific antibody response. A: C57BL/6 mice were tolerised with either 100 µg GP61-80-IFA, 100 µg NP309-328-IFA, or both peptides in IFA on days -109, -106, -103. On day 0, mice were infected with 200pfu LCMV-WE and splenocytes were analysed for intracellular IFN-γ expression after re-stimulation with different T cell epitopes *in vitro* 12 days later. The original dot plots are shown (n = 5). B: C57BL/6 mice were treated with 100 µg GP61-80-IFA or IFA on days -9, -6 and -3. On day 0, mice were infected with 200pfu LCMV-WE and splenocytes were analysed for intracellular IFN-γ after re-stimulation with different T cell epitopes *in vitro* 12 days later (n = 3, p<0.001). C+D: C57BL/6 mice were tolerized with either 100 µg GP61-80-IFA, 100 µg NP309-328-IFA or both peptides in IFA on days -9, -6, -3. On day 0, mice were infected with 200pfu LCMV-WE and LCMV-nucleoprotein (NP) specific antibodies were measured by ELISA at the indicated time points (n = 5, * p<0.05, C). LCMV-glycoprotein (GP) specific antibodies were measured by ELISA at the indicated time points (n = 5–7, *p<0.001, D). E: BALB/c mice were peptide-tolerised with 100 µg GP61-80-IFA or IFA alone on day -9, -6 and -3. On day 0 mice were infected with 200pfu of LCMV-WE and LCMV-GP specific IgG antibodies were measured in the serum at the indicated time points (n = 8, ns).

Next, we analyzed whether peptide tolerisation of the LCMV glycoprotein-derived epitope (GP61) or the LCMV nucleoprotein derived NP309 epitope influenced protective antibody responses. Following specific peptide treatment and LCMV infection of C57BL/6 mice, endogenous CD4^+^ T cells specific for both the immunodominant T helper epitope from the LCMV-glycoprotein GP61-80 (GP61) and for the LCMV-nucleoprotein NP309-328 (NP309, Figure 2A) were rendered unresponsive. This non-responsiveness of endogenous CD4^+^ T cells was long lasting, as *in vitro* GP61 peptide re-stimulation of GP61-IFA treated LCMV infected mice after 100 days showed virtually no expansion of GP61-specific T cells (Figure 2B). Serum of such treated C57BL/6 mice was then analyzed for antibody formation. Tolerisation of the NP309-328 T helper cell response did not significantly affect the antibody response against LCMV-glycoprotein. In contrast, the antibody response against the LCMV-glycoprotein GP1 portion carrying the neutralizing epitope [24] was clearly accelerated and of higher titer after tolerisation with GP61-80 alone or with GP61-80 plus NP309-328 (Figure 2C+D). Antibody responses directed to LCMV-nucleoprotein were only slightly enhanced after tolerisation with these peptides (Figure 2C). As the precursor frequency of NP-specific B cells is high in naïve mice, antibody responses against LCMV-NP are already quite strong without tolerisation [24]. Therefore the limited B cell repertoire directed against GP1 [24] could benefit most from tolerisation. To demonstrate in a secondary manner that the enhanced antibody response was indeed a consequence of the altered T helper cell response and not of direct B cell stimulation, we peptide-tolerised H-2^d^ BALB/c mice which do not present the GP61-80 epitope on MHC II. In contrast to the clearly enhanced GP-1-specific antibody responses in peptide-tolerised C57BL/6 mice, we found no difference in GP1-specific IgG antibodies in GP61-80 peptide-treated or peptide non-treated BALB/c mice (Figure 2E).

### Maintained CD8^+^ T cell function is maintained after specific CD4^+^ T cell tolerisation

**Figure 3:**
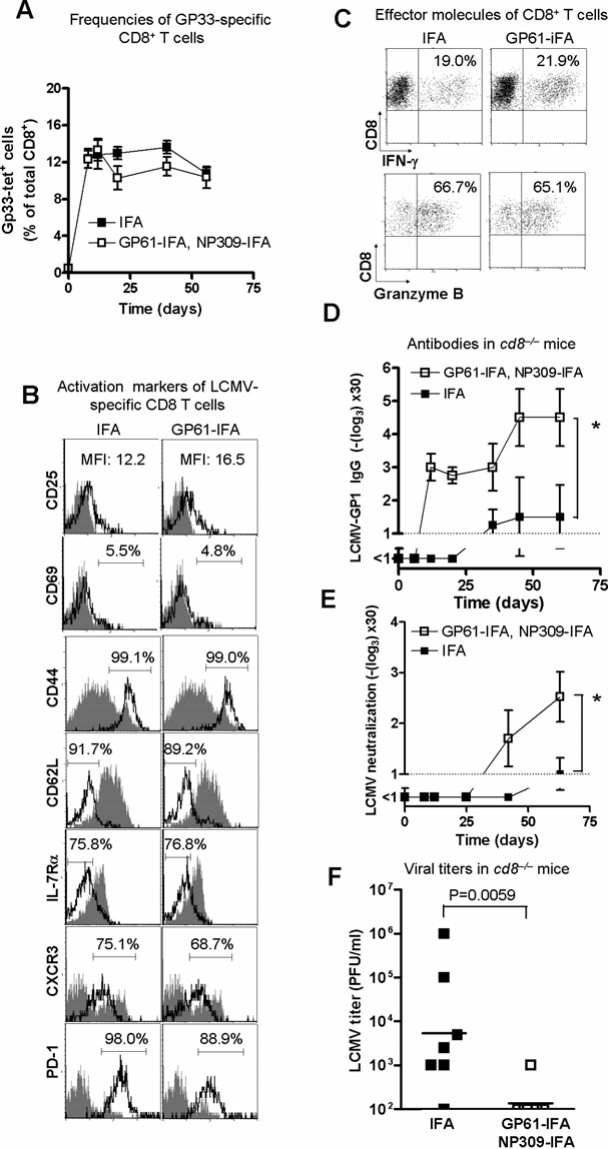
Peptide tolerisation improves protective antibody responses independently and without impairment of CD8^+^ T cells. A: C57BL/6 mice were treated with GP61-IFA and NP309-IFA, while control mice were treated with IFA alone at days -9, -6, -3. At day 0 mice were infected with 200pfu LCMV-WE, and GP-specific (tet-gp33^+^) CD8^+^ T cells were analyzed in the blood by FACS analysis (n = 4, ns). B+C: 5×10^4^ splenocytes from mice transgenic for a T cell receptor recognizing the LCMV helper epitope GP61 (LCMV-glycoprotein_61-80_/I-A^b^-specific TCR, SMARTA mice) and for the T cell marker Thy1.1 were transferred into C57BL/6 mice on day -10. One group of mice was treated with 100 µg GP61 dissolved in IFA, while control mice were treated with IFA alone at days -9, -6, -3. At day 0 mice were infected with 200pfu LCMV-WE or left untreated. GP33 specific CD8^+^ T cells were analyzed for frequencies (Figure S3) and for the expression of the activation markers CD25, CD69, CD44, CD62L, IL-7Rα, CXCR3 and PD-1 (1 of 4 histogram plots is shown, grey area represents staining of naïve CD8^+^ T cells, B). CD8^+^ T cells were analyzed for expression of the intracellular effector molecules IFN-γ and Granzyme B after restimulation (1 of 4 dot plots is shown, C). D–F: *cd8^−/−^* mice were treated with GP61/NP309-IFA or with IFA alone on days -9, -6 and -3. On day 0 mice were infected with 200pfu LCMV-WE and LCMV-GP1 specific IgG antibodies were detected by ELISA (n = 3–4, *p = 0.03, D) and by neutralisation assay (n = 7–8, *p = 0.04, E) at the indicated time points. Virus blood titers were measured with plaque assay on day 35 after LCMV infection as indicated (n = 7–8, p = 0.0059, F).

In various infection models it has been documented that CD4^+^ T cell help is important for CD8^+^ T cell function and especially CD8^+^ T cell memory [25]–[27]. We found no differences in LCMV-specific gp33-tet^+^ CD8^+^ T cells between tolerised and non-tolerised mice during infection with 200 pfu LCMV-WE (Figure 3A). During infection with 2×10^6 ^pfu LCMV-WE, overwhelming replication of LCMV leads to partial deletion of the NP396 epitope-specific CD8 T cell repertoire (Figure S3). Infection of GP61-IFA tolerised mice with 2×10^6 ^pfu led to normal expansion of tet-gp33^+^ cells. Mice treated with GP61-IFA had similar frequencies of tet-gp33^+^ CD8^+^ T cells after high dose LCMV infection as IFA only treated mice suggesting that tolerisation does not influence exhaustion of CD8^+^ T cells (Figure S3). For more precise analysis of CD4^+^ T cell tolerisation-derived effects on CD8^+^ T cell function upon peptide tolerisation, 5×10^4^ splenocytes from SMARTA mice were transfused into C57BL/6 mice on day -10. One group of mice was treated with 100 µg GP61 emulsified in IFA, while control mice were treated with IFA alone at days -9, -6, -3. At day 0, mice were infected with 200pfu LCMV-WE or left untreated. Seven days after infection, the frequency of LCMV-specific (GP33-specific) CD8^+^ T cells was analyzed by tetramer staining in the spleen. No difference was observed in overall CD8^+^ T cell frequencies with or without T helper cell tolerisation (Figure S4). CD8^+^ T cells showed no difference neither in activation markers CD25, CD69, CD44, CD62L, IL-7Rα, CXCR3 nor PD-1 (Figure 3B). Consistent with these results, no differences were found in expression of intracellular IFN-γ and Granzyme B in CD8^+^ T cells after GP33 re-stimulation (Figure 3C). These results rendered it unlikely that the enhanced antibody production following helper T cell tolerisation was due to a difference in the primary CD8^+^ T cell response. Since virus elimination after infection with 200pfu LCMV is known to be mediated by CD8^+^ T cells [28], differences in the amount of viral antigen could not be responsible for the measured differences in antibody formation. Nevertheless, we also evaluated whether GP61 or NP309 tolerisation enhanced the LCMV-GP specific antibody response in *cd8^−/−^* mice. In line with the previous findings in C57BL/6 mice, an earlier and higher GP-specific IgG antibody response was found in GP61/NP309-IFA tolerised *cd8^−/−^* mice (Figure 3D). Higher LCMV GP-1 specific IgG titers measured by ELISA also correlated with earlier and higher LCMV neutralizing antibody titers as measured by a virus neutralisation assay (Figure 3E). By day 35, virus was undetectable in blood of 7 out of 8 GP61/NP309-IFA treated *cd8^−/−^* mice while it was controlled in only 1 of 7 IFA-treated mice (Figure 3F, p = 0.0059). As an additional control, the observed virus titer differences of peptide treated *vs* peptide non-treated mice in the absence of CD8^+^ T cells was not found in LCMV-infected, CD8^+^ T cell-depleted, B cell-deficient *jh^−/−^* mice. This further supported that virus control was B cell-dependent in CD8^+^ T cell deficient mice (Figure S5). In conclusion these data indicated that tolerisation with GP61-IFA enhanced protective antibodies independently of CD8^+^ T cells.

### Protective antibody responses against a “third-party” virus are not affected by GP61-IFA tolerization

**Figure 4:**
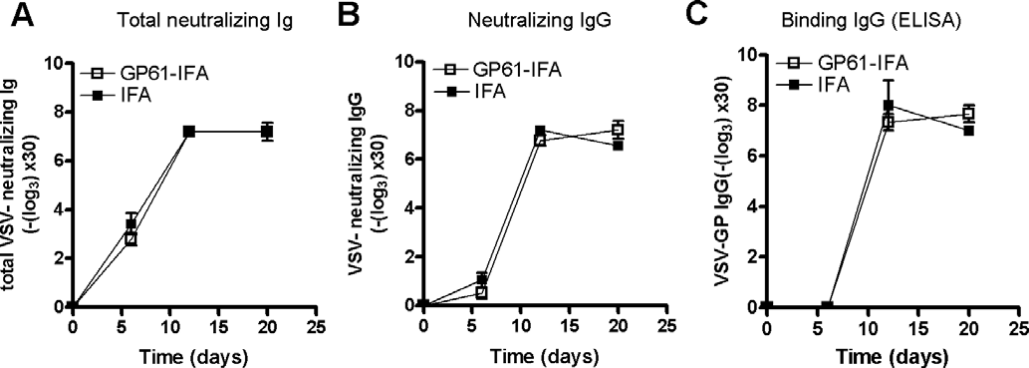
LCMV-GP specific peptide tolerisation does not affect antibody production against third party pathogen. C57BL/6 were tolerised with 100 µg GP61-IFA at day -9, -6 and -3. At day 0, mice were infected with 2×10^6^ PFU of vesicular stomatitis virus (VSV). Neutralizing antibodies against VSV-glycoprotein were measured by VSV neutralisation assay; total neutralizing Ig (A) and neutralizing IgG (B). VSV-glycoprotein binding IgG was measured by ELISA (C).

Lastly, we analysed whether tolerisation with GP61 peptide generally impaired CD4^+^ T cell function in response to a third-party pathogen. C57BL/6 mice were tolerised with GP61-80 peptide and were then infected with vesicular stomatitis virus (VSV). Following VSV infection, a rapid CD4^+^ T cell-dependent IgG formation is necessary to prevent lethal encephalitis [29]. Treatment of C57BL/6 mice with GP61-IFA affected neither the anti-VSV IgM nor the IgG response (Figure 4).

## Discussion

Peptide given i.p. in incomplete Freund̀s Adjuvant lead to unresponsiveness of specific CD4^+^ T cells. Interestingly, subcutaneous application of peptide in complete Freund̀s Adjuvant or in incomplete Freund̀s Adjuvant leads to priming of immune cells [30]. This suggests that systemic persistence rather than the stimulatory quality of IFA is responsible for induction of unresponsiveness. The unresponsive phenotype of CD4^+^ T cells was characterised by a lack of proliferation and a lack of IFN-γ production even after restimulation with PMA and ionomycin, which excludes anergy as a potential mechanism of CD4^+^ T cell unresponsiveness. We observed small differences in differentiation markers on tolerised CD4^+^ T cells, which might hint to altered differentiation of non-responsive T cells.

T cell tolerisation augmented the usually very late protective antibody response against the non-cytopathic persistence-prone virus LCMV. It is not clear so far how exactly T cell tolerisation influences antibody responses. However, together with the data derived from our former study [14] we speculate that an overwhelming CD4^+^ T helper cell response will preferentially trigger low affinity non protective B cells. Reduction of CD4 T cell help will therefore shift the balance of switched B cells to more high affinity B cells.

Since this tolerisation procedure is in contrast to conventional vaccination strategies aiming at an enhancement of immune responses, we named it “negative vaccination”. The primary specific CD8^+^ T cell responses remained unaffected by peptide tolerisation. Although we have not directly analysed memory CD8^+^ T cell responses in T helper cell peptide-tolerized mice, we believe them to be unaffected due to two reasons. First, the T helper cell support to CD8^+^ T cell memory [25] has been demonstrated to be antigen non-specific [31]. Second, partial T helper cell depletion did not reduce memory CD8^+^ T cell responses as tested in an *in vivo* cytotoxicity assay 150 days following infection [14].

It is difficult to rapidly induce neutralizing antibodies against surface glycoproteins of several persistent pathogens in man, including HCV, HIV or several parasitic infections, where neutralizing antibodies become detectable often only after 50–200 days, if at all. Our results here raise the obvious question of whether similar antibody-suppressive effects of T helper cells may be important in HIV or HCV infection and whether reduction of specific CD4^+^ T cell help may accelerate and augment HCV- or HIV-neutralizing antibody responses. As it is not possible to work with HIV or HCV in mice this topic is not easy to address. Analysing other infection models might give some more insights about the role of CD4 T helper cell tolerisation on antibody responses during infection with persistence prone viruses, however this still is not representative for HIV or HCV infection in humans. However, there are some correlative findings about the role of strong CD4 T cell help and neutralizing antibodies in human persistent prone infections. It was demonstrated that strong T helper cell responses are associated with low neutralizing antibodies after HCV infection in man, although they were associated with virus clearance in this study [32]. However, a study examining spontaneous HCV clearance in young patients did measure unexpected low helper T cell responses [33]. This is consistent with another study that could not find a correlation of the T cell response and HCV clearance [34].

In addition, experiments to improve virus-specific T helper cells before SIV challenge in macaques have resulted in paradoxically higher virus load and more rapid death of animals [35]. Recent experiments with SIV have also demonstrated that generally reduced cellular immune responses may be associated with enhanced survival [35]. This correlates with the finding that high T helper cell numbers before HIV infection are an independent risk factor for more rapid disease progression [36]. As CD4^+^ T cells are themselves a target of HIV, those data are difficult to interpret and more studies have to be done to rule out a potential role of CD4 T cell tolerisation on the impact of neutralizing antibodies and outcome of disease.

In conclusion, this new “negative vaccination” protocol to tolerise virus-specific CD4^+^ T cells leads to an efficient and selective enhancement of virus-specific protective antibody responses during LCMV infection, leaving third-party CD4^+^ T cell responses unaffected. It may offer a new strategy for depletion of overwhelming CD4^+^ T cell help in human disease.

## Materials and Methods

### Mice and viruses

LCMV strain WE was originally obtained from F. Lehmann-Grube (Heinrich Pette Institute, Hamburg, Germany) and was propagated in L929 cells. LCMV and VSV neutralization assays were performed as described [14]. Virus blood titers were measured using a plaque forming assay as described [14]. Mice were infected with 200 plaque forming units (pfu) LCMV-WE. Mice transgenic for a T cell receptor recognizing LCMV glycoprotein_61-80 _(LCMV-GP61/I-A^b^-specific TCR, SMARTA mice) were maintained on the C57BL/6 genetic background. All experiments were performed in single ventilated cages. Animal experiments were carried out with authorization of the Veterinäramt of the Kanton Zurich and in accordance with the Swiss law for animal protection.

### Preparation of peptide in incomplete Freund Adjuvant (IFA)

GP61 (GP61-80, GLNGPDIYKGVYQFKSVEFD) or NP309 (NP309-328, SGEGWPYIACRTSVVGRAWE) were synthesized by Neosystems. Peptides were dissolved in DMSO in 50 µg/µl and than diluted to 1 µg/µl in PBS. Peptide dissolved in PBS was mixed 1∶1 with incomplete Freund Adjuvant (IFA, DIFCO Laboratories, Detroit Michigan 48232-7038, USA). As control DMSO diluted in PBS was mixed with IFA 1∶1.

### FACS analysis

Tetramer production and FACS analysis was performed as described previously [17]. Briefly, splenocytes or peripheral blood lymphocytes were stained using PE-labeled GP33 MHC class I tetramers (GP33/H-2D^b^) for 15 minutes at 37°C, followed by staining with anti-CD8 (BD Biosciences) for 30 minutes at 4°C. For determination of LCMV specific CD4^+^ T cells lymphocytes were stained with anti-CD4 and anti Thy1.1 (CD90.1, BD Biosciences). For determination of their activation status, lymphocytes were stained with anti-CD25, anti-CD69, anti GITR, anti-CD62L, anti-CD44 and anti IL-7Rα (BD Biosciences) for 30 minutes at 4°C. For cytokine analysis cells were re-stimulated in vitro with antigen or with PMA/Ionomycin and fixed with 1% Formalin. Cells were permeabilized with saponin and stained for intracellular IFN-γ, IL-10, IL-4 (BD Biosiences) and intracellular Granzyme B (CALTAG, Burlingame, CA). For staining of regulatory T cells, an anti-FoxP3 kit was used (eBiosciences).

### ELISA

96-well plates were coated with 100 µl recombinant LCMV-glycoprotein-GP1-supernatant[14], baculo LCMV-NP or baculo VSV-GP. 1∶30 pre-diluted sera were incubated for 90 min, washed with PBS containing 0.5% Tween 20, then incubated further with anti-mouse IgG-horseradish-peroxidase (HRP) (1∶1000) (Sigma-Aldrich) for detection of virus-specific IgG responses. For the detection of IgG antibodies specific for LCMV GP, plates were coated with anti human Fc, followed by incubation with the LCMV-GP1-Fc[14]. Plates were then blocked with 2% bovine serum albumin (in PBS) for 2 h at room temperature. 1∶30 pre-diluted sera were incubated for 90 min. Anti mouse-IgG-HRP (1∶1000) (Sigma-Aldrich) was used to detect specific IgG-responses. In all ELISA-assays a green colour-reaction was produced using 2.2′-azino-bis(3-ethylbenzothiazoline-6-sulfonate (ABTS) (Boehringer-Mannheim) as a substrate. The ELISA-titer was defined as the log_2_-serum-dilution resulting in an optical density (OD_405_) two-fold above background.

**Neutralization assay** was performed as previously described [14]. Sera were pre-diluted 30× or 40× and than titrated with a dilution step of 2×. To compare the neutralization titers with data derived from ELISA, results were calculated, and presented as (log3)×30.

### Statistical analysis

Data are expressed as mean±S.E.M. Statistical significant differences between two different groups were analyzed using students t test. Analysis including several groups were tested with one-way ANOVA with additional Bonferoni or Dunnett test. Statistical significant differences between treatment groups which were analyzed to several time points were analyzed using two-way ANOVA (repeated measurements). p values<0.05 were considered as statistically significant.

## Supporting Information

Figure S1 5×104 splenocytes from mice transgenic for a T cell receptor recognizing the LCMV helper epitope GP61 (LCMV-glycoprotein61-80/I-Ab-specific TCR, SMARTA mice) and expressing the T cell marker Thy1.1 were transferred into C57BL/6 mice on day -10. One group of mice was treated with 100µg GP61 disolved in IFA, while control mice were treated with IFA alone at days -9, -6, -3. At day 0 mice were infected with 200pfu LCMV-WE or left untreated. Seven days after infection GP61-specific Thy1.1+ CD4+ T cells and CD4+ T cells from untreated B6 mice were analyzed for expression of GITR, PD-1 and regulatory T cells by CD25 and FoxP3 staining.
(0.41 MB TIF)

Figure S2 Shows transfer scheme from experiment presented in Figure 1H.
(0.41 MB TIF)

Figure S3 C57BL/6 mice were treated with 100microg GP61 dissolved in IFA, while control mice were treated with IFA alone at days -9, -6, -3. At day 0 mice were infected with 2×106pfu LCMV-WE. GP33 and NP396 specific CD8+ T cells were analyzed in the blood on day 12 after infection.
(0.38 MB TIF)

Figure S4 5×104 splenocytes from mice transgenic for a T cell receptor recognizing the LCMV helper epitope GP61 (LCMV-glycoprotein61-80/I-Ab-specific TCR, SMARTA mice) and for the T cell marker Thy1.1 were transferred into C57BL/6 mice on day -10. One group of mice was treated with 100µg GP61 dissolved in IFA, while control mice were treated with IFA alone at days -9, -6, -3. At day 0 mice were infected with 200pfu LCMV-WE or left untreated. GP33 specific CD8+ T cells were analyzed for frequencies.
(0.38 MB TIF)

Figure S5 Jh-/- mice were treated with 100 µg GP61 dissolved in IFA or with IFA alone at days -9, -6, -3. On days -2 and -1 CD8 T cells were depleted. At day 0 mice were infected with 200pfu LCMV-WE. Mice were analyzed for replicating virus in the blood at the indicated time points.
(0.38 MB TIF)
