# Supplementary figures and images for: “Negative Vaccination” by Specific CD4+ T Cell Tolerisation Enhances Virus-Specific Protective Antibody Responses
Source: PLoS One. 2007 Nov 14;2(11):e1162. doi: 10.1371/journal.pone.0001162 (PMC2048666; doi:10.1371/journal.pone.0001162)

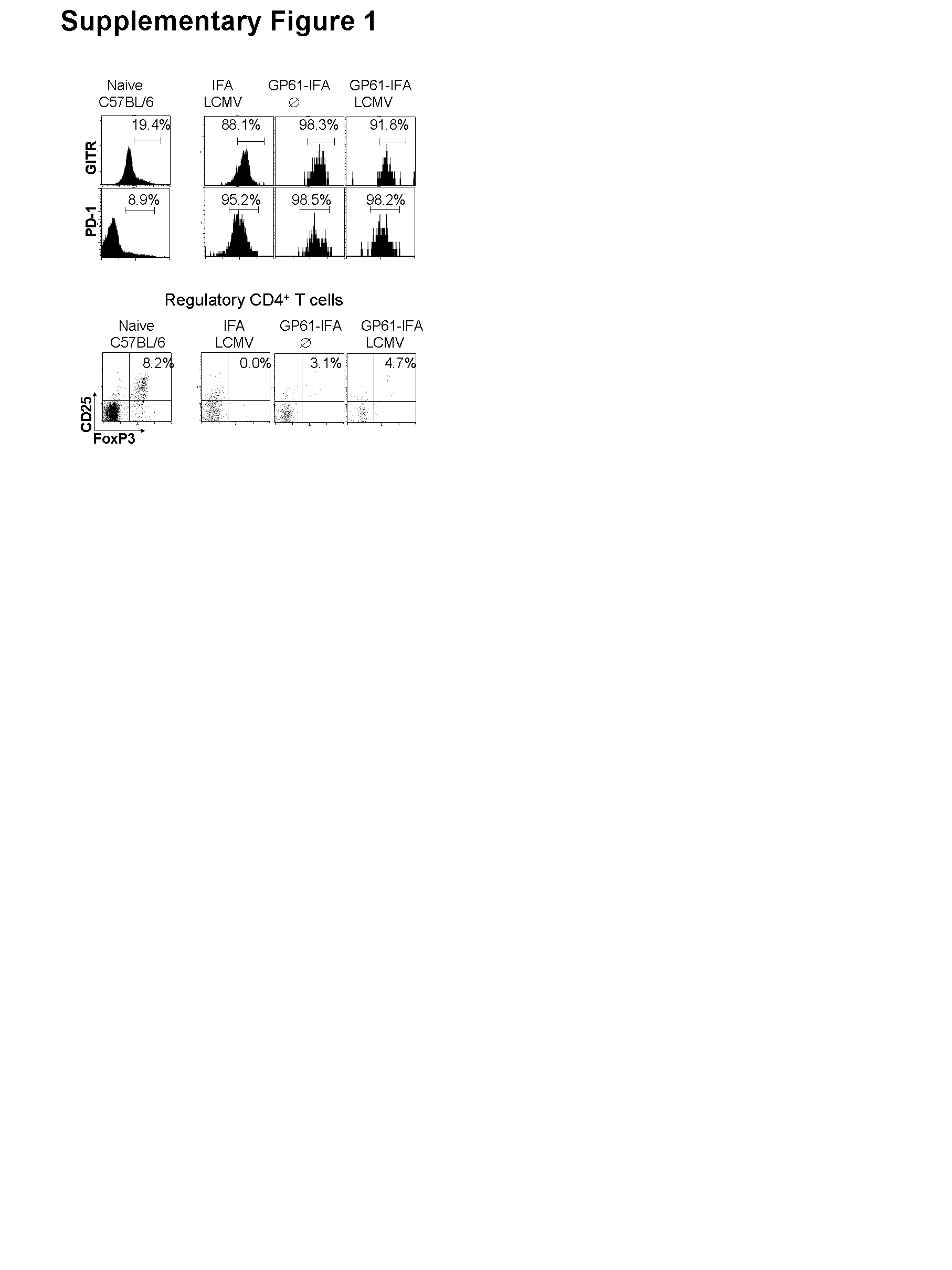

Supplement: Figure S1 — 5×10^4^ splenocytes from mice transgenic for a T cell receptor recognizing the LCMV helper epitope GP61 (LCMV-glycoprotein61-80/I-Ab-specific TCR, SMARTA mice) and expressing the T cell marker Thy1.1 were transferred into C57BL/6 mice on day -10. One group of mice was treated with 100µg GP61 disolved in IFA, while control mice were treated with IFA alone at days -9, -6, -3. At day 0 mice were infected with 200pfu LCMV-WE or left untreated. Seven days after infection GP61-specific Thy1.1+ CD4+ T cells and CD4+ T cells from untreated B6 mice were analyzed for expression of GITR, PD-1 and regulatory T cells by CD25 and FoxP3 staining. (0.41 MB TIF) [file pone.0001162.s001.tif]

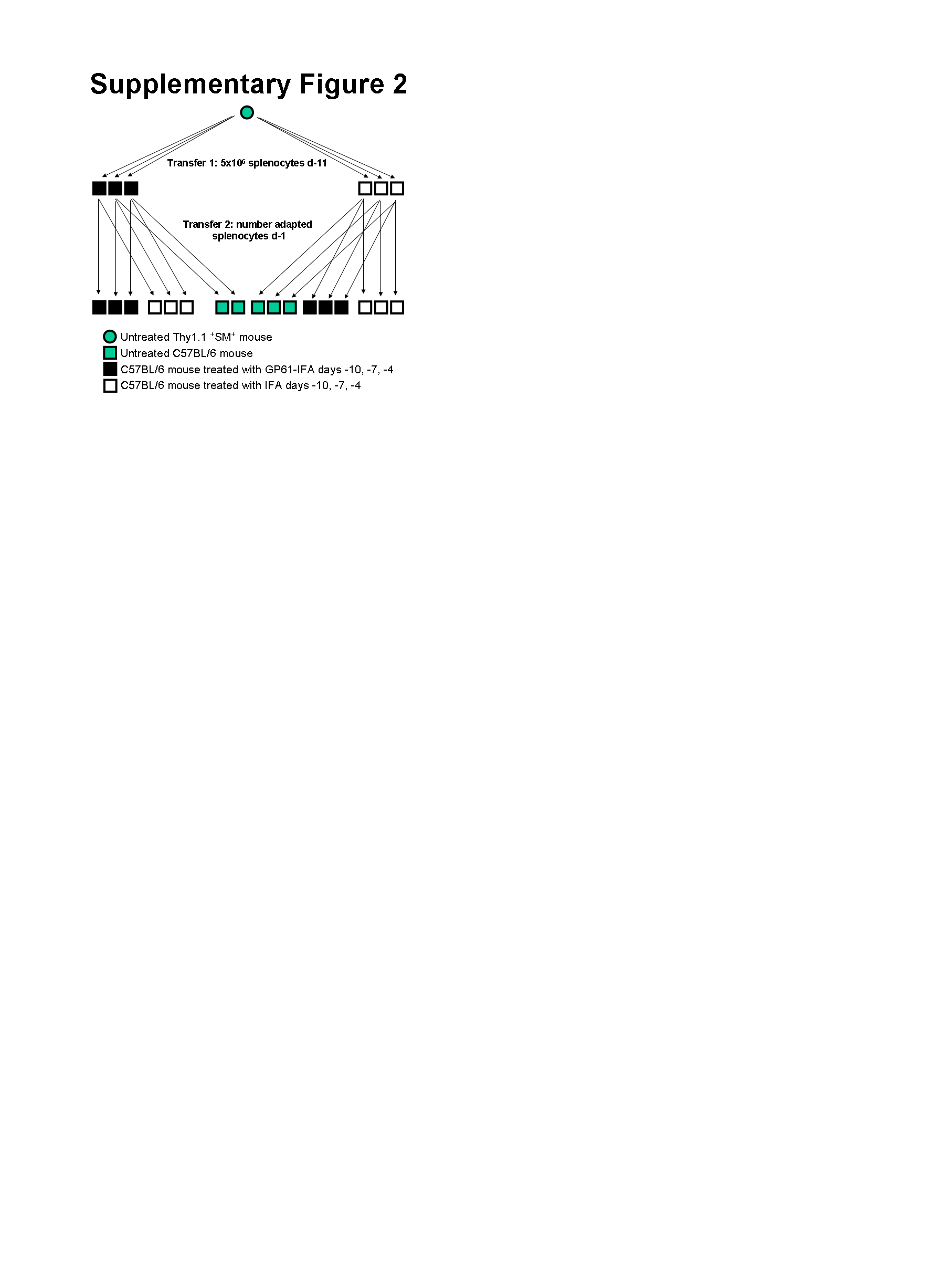

Supplement: Figure S2 — Shows transfer scheme from experiment presented in Figure 1H. (0.41 MB TIF) [file pone.0001162.s002.tif]

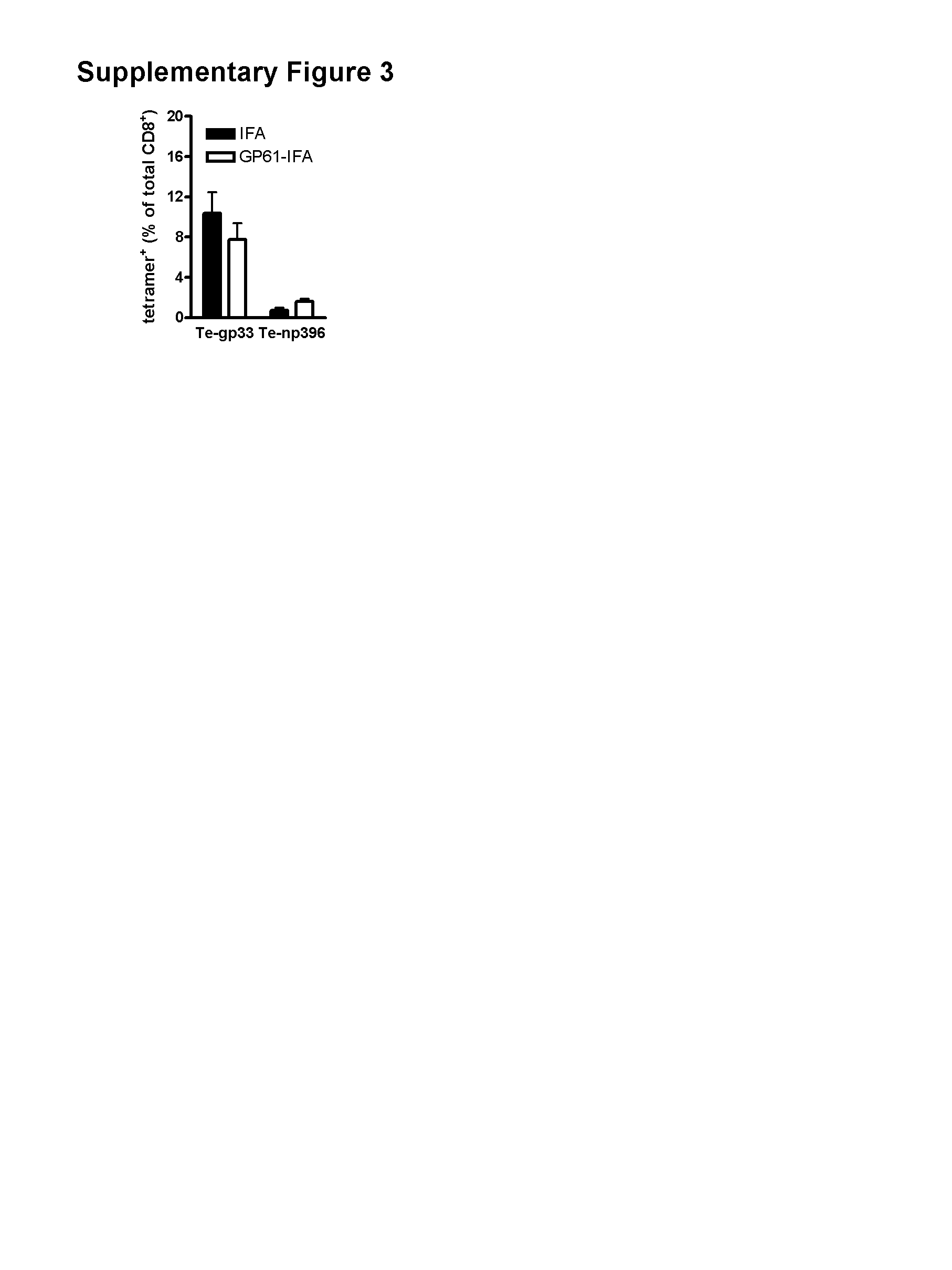

Supplement: Figure S3 — C57BL/6 mice were treated with 100microg GP61 dissolved in IFA, while control mice were treated with IFA alone at days -9, -6, -3. At day 0 mice were infected with 2×10^6^pfu LCMV-WE. GP33 and NP396 specific CD8+ T cells were analyzed in the blood on day 12 after infection. (0.38 MB TIF) [file pone.0001162.s003.tif]

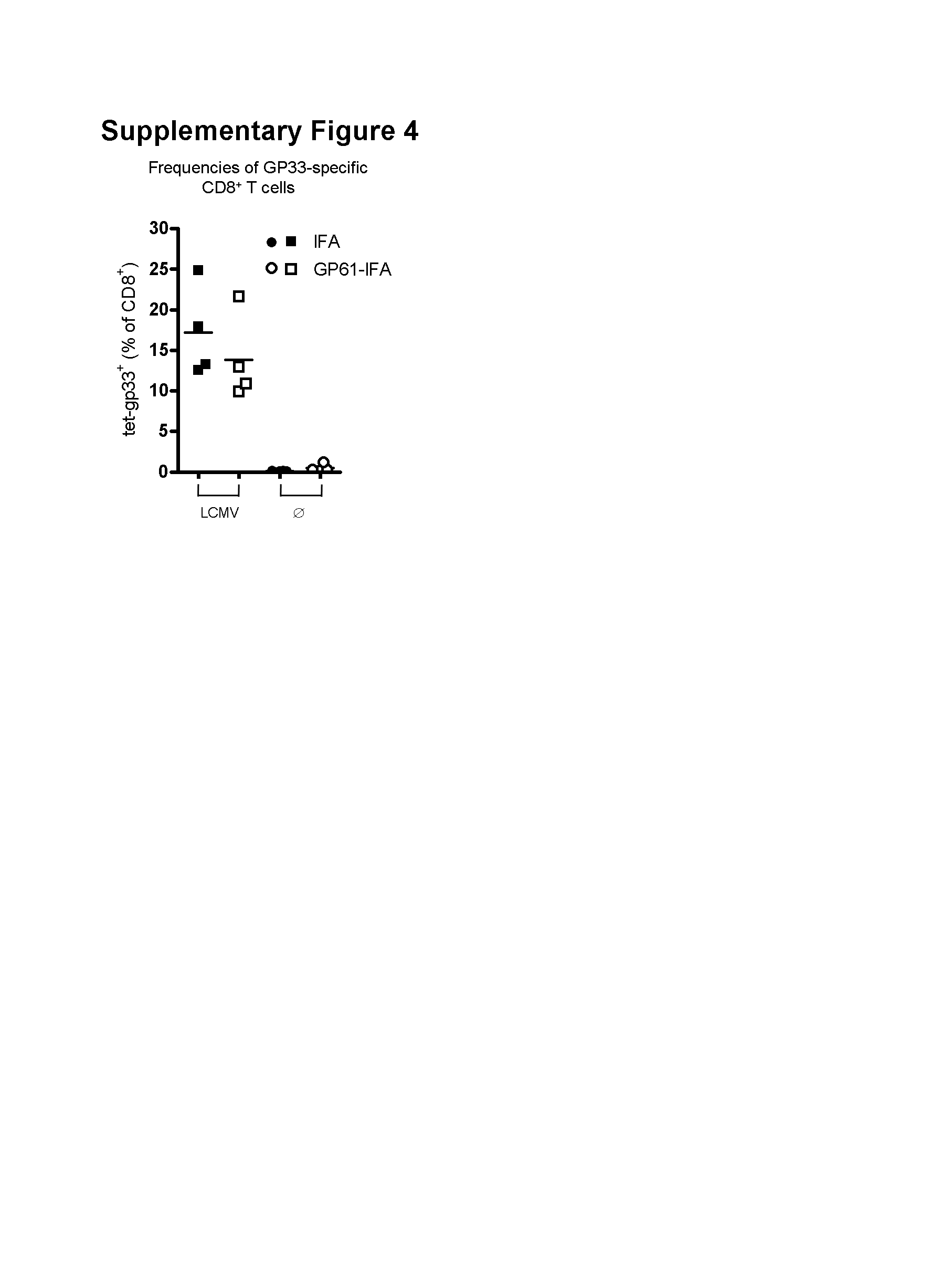

Supplement: Figure S4 — 5×10^4^ splenocytes from mice transgenic for a T cell receptor recognizing the LCMV helper epitope GP61 (LCMV-glycoprotein61-80/I-Ab-specific TCR, SMARTA mice) and for the T cell marker Thy1.1 were transferred into C57BL/6 mice on day -10. One group of mice was treated with 100µg GP61 dissolved in IFA, while control mice were treated with IFA alone at days -9, -6, -3. At day 0 mice were infected with 200pfu LCMV-WE or left untreated. GP33 specific CD8+ T cells were analyzed for frequencies. (0.38 MB TIF) [file pone.0001162.s004.tif]

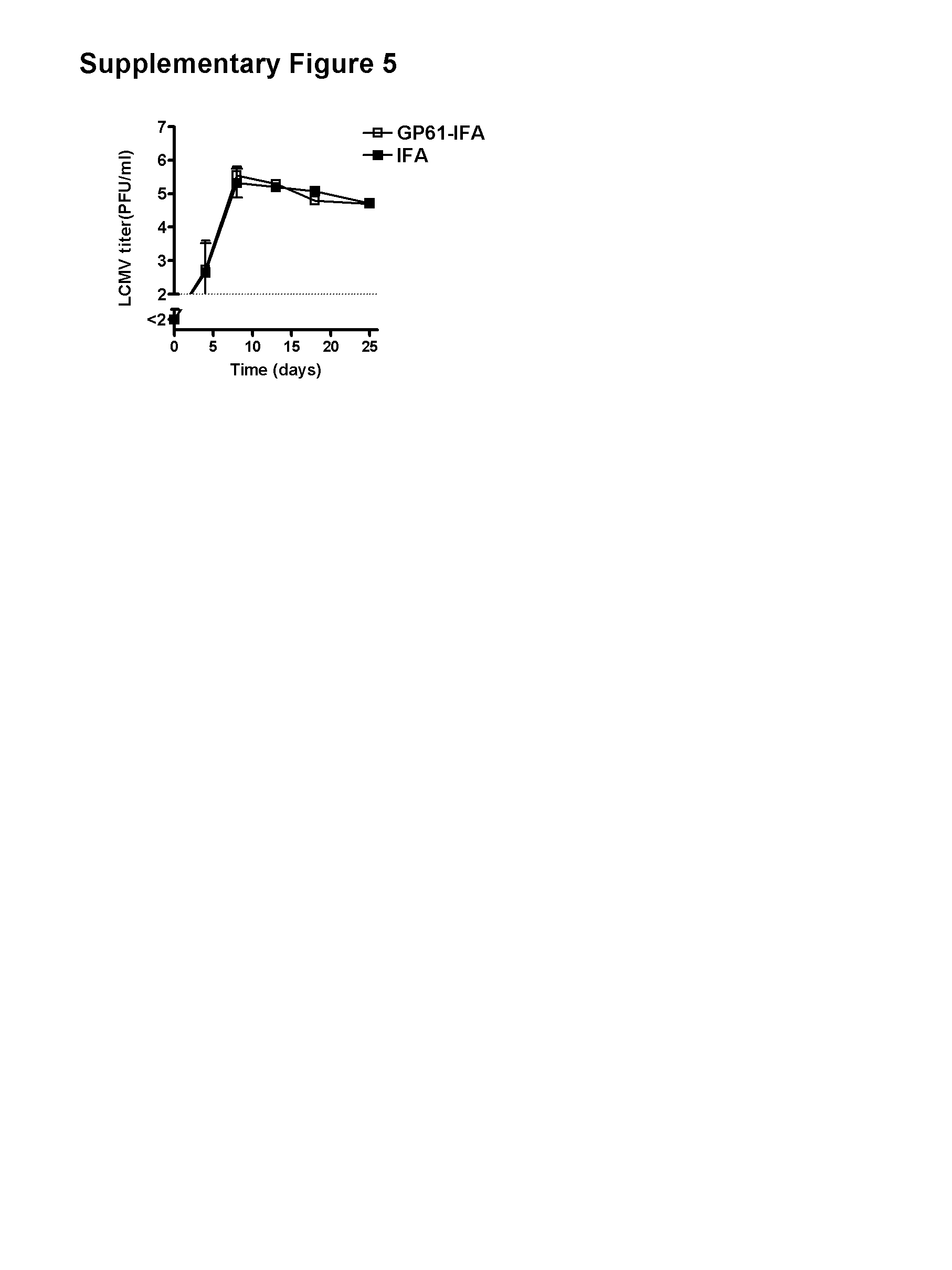

Supplement: Figure S5 — Jh-/- mice were treated with 100 µg GP61 dissolved in IFA or with IFA alone at days -9, -6, -3. On days -2 and -1 CD8 T cells were depleted. At day 0 mice were infected with 200pfu LCMV-WE. Mice were analyzed for replicating virus in the blood at the indicated time points. (0.38 MB TIF) [file pone.0001162.s005.tif]
